# The Development and Public Health Implications of Food Preferences in Children

**DOI:** 10.3389/fnut.2017.00066

**Published:** 2017-12-18

**Authors:** Jacob P. Beckerman, Queen Alike, Erika Lovin, Martha Tamez, Josiemer Mattei

**Affiliations:** ^1^Department of Nutrition, Harvard T.H. Chan School of Public Health, Boston, MA, United States; ^2^Department of Social and Behavioral Sciences, Harvard T.H. Chan School of Public Health, Boston, MA, United States; ^3^Harvard Kennedy School of Government, Cambridge, MA, United States; ^4^Wharton School of the University of Pennsylvania, Philadelphia, PA, United States

**Keywords:** food preferences, eating behaviors, healthy food choices, taste development, feeding practices

## Abstract

Food preferences are a primary determinant of dietary intake and behaviors, and they persist from early childhood into later life. As such, establishing preferences for healthy foods from a young age is a promising approach to improving diet quality, a leading contributor to cardiometabolic health. This narrative review first describes the critical period for food preference development starting *in utero* and continuing through early childhood. Infants’ innate aversion to sour and bitter tastes can lead them to initially reject some healthy foods such as vegetables. Infants can learn to like these foods through exposures to their flavors *in utero* and through breastmilk. As solid foods are introduced through toddlerhood, children’s food preferences are shaped by parent feeding practices and environmental factors such as food advertising. Next, we discuss two key focus areas to improve diet quality highlighted by the current understanding of food preferences: (1) promoting healthy food preferences through breastfeeding and early exposures to healthy foods and (2) limiting the extent to which innate preferences for sweet and salty tastes lead to poor diet quality. We use an ecological framework to summarize potential points of intervention and provide recommendations for these focus areas, such as worksite benefits that promote breastfeeding, and changes in food retail and service environments. Individuals’ choices around breastfeeding and diet may ultimately be influenced by policy and community-level factors. It is thus crucial to take a multilevel approach to establish healthy food preferences from a young age, which have the potential to translate into lifelong healthy diet.

## Introduction

Food preferences begin taking shape during fetal development and continue changing throughout life, influenced by biological, social, and environmental factors (1, 2). These preferences are key determinants of food choices, and therefore diet quality (2, 3). Diets low in fruits and vegetables are estimated to account for 4.2 and 1.5% of global disability-adjusted life years, respectively (4).

**Figure 1:**
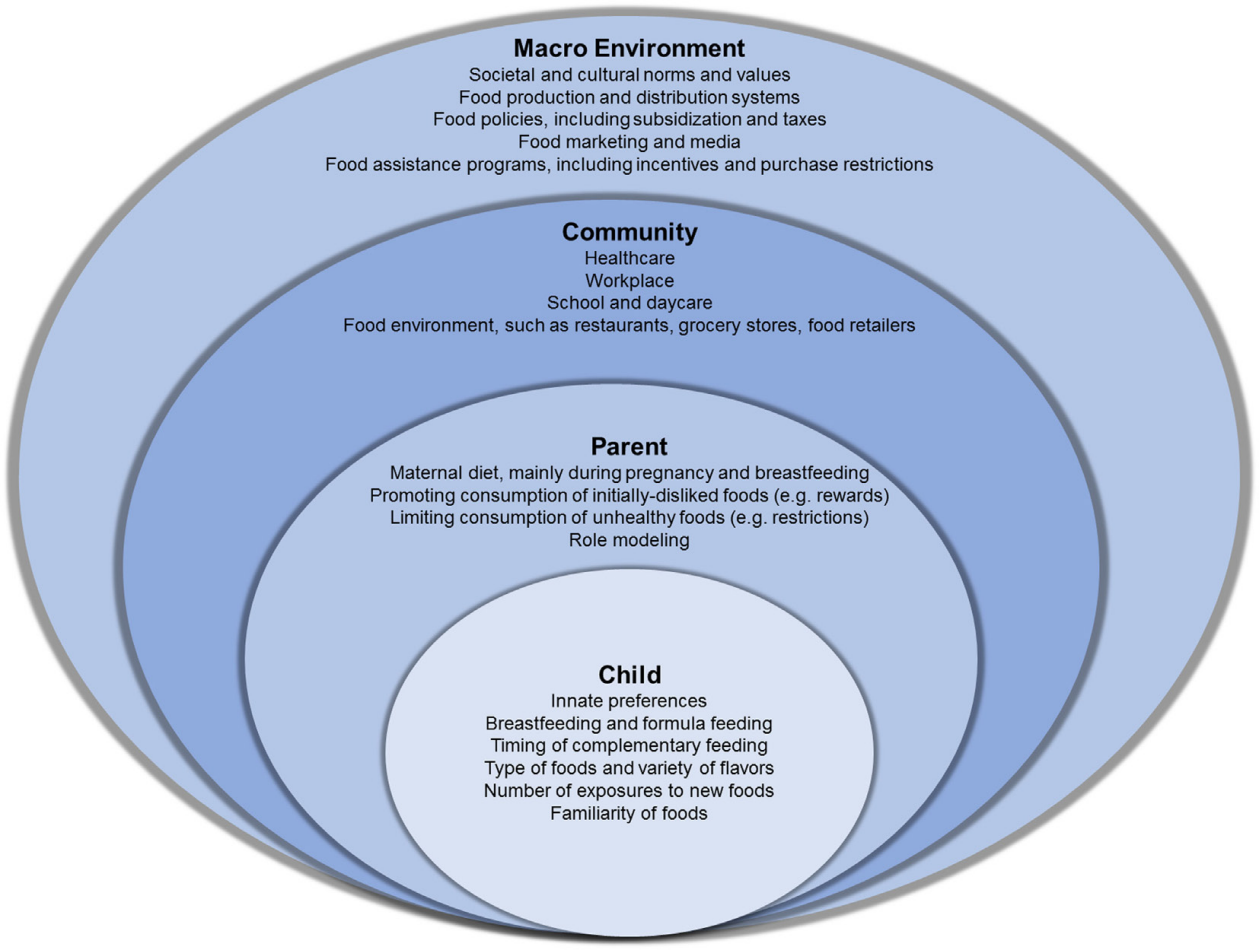
An ecological model of the influences on food preferences in children. The child and parent levels describe known factors that shape food preferences in children, starting at birth. The community and macro-environment include factors with known direct effects on food preferences as well as factors that may indirectly influence food preferences through their effects on individual behaviors.

Early childhood is a critical period to establish food preferences (1), making it an ideal age for efforts to improve diet quality. Furthermore, investments made during this period will be compounded over time, as food preferences established in early childhood persist into later life (1, 5). We begin this narrative review by summarizing current knowledge on the development of food preferences in children starting *in utero*. We then discuss the potential of breastfeeding and nutrition policies and programs to improve nutrition by shaping food preferences using an ecological framework (Figure 1). We conclude by proposing recommendations to promote healthy food preferences.

## Influences on Food Preferences

### Pre- and Postnatal Influences

Infants have innate preferences for sweet, salty, and umami tastes while they reject sour and bitter tastes, which may help them consume energy- and protein-dense foods while avoiding potentially toxic foods (1, 5, 6). Infants’ innate tendencies may lead them to reject some healthy foods such as bitter-tasting vegetables (5). Genetic determinants influence food preferences (1, 7), yet they are beyond the scope of this review. Children can learn to like the flavors of foods by being exposed to them, which begins *in utero* and continues during breastfeeding and formula feeding (1, 5, 6). Fetuses are able to detect changing odors in the amniotic fluid by 11 weeks (8), allowing them to perceive flavors that come from foods mothers eat (1, 5, 6). Similarly, flavors of the maternal diet during nursing are present in breastmilk (1, 5, 6). These pre- and postnatal flavor exposures may influence a child’s preferences for those flavors later in infancy (1, 5, 6). For example, 5- to 8-month-old infants of mothers randomized to drink carrot juice during the third trimester of pregnancy (9) or during lactation (9, 10) had less aversion to carrot-flavored cereal than children of mothers who only drank water. While breastfed infants are generally more accepting of novel flavors than formula-fed infants (6, 11), the control groups in Mennella et al.’s randomized trials (9, 10) were also breastfed, so the authors concluded that the increased acceptance was due to exposure through breastmilk. Notably, null associations between breastfeeding duration and infants’ acceptance of fruits and vegetables during the first 2 months of weaning have been reported (12). It is unclear how many flavor exposures through breastmilk are required to impact infant acceptance of a flavor; maternal consumption of caraway-flavored hummus 10 times during nursing did not affect breastfed infants’ later acceptance of caraway flavor (13). Formula-fed infants also learn to prefer the flavors to which they are exposed; those fed soy-based or hydrolyzate-based formula prefer the specific flavors in these formulas (1, 5). Breastfeeding introduces the infant to a wider variety of flavors than formula, which may explain why breastfed children have more diverse food preferences upon the introduction of complementary foods (6). Although simple exposure is not the only learning mechanism that can modify food preferences, it plays a central role in pre- and postnatal flavor learning, and thus the quality of the mother’s diet becomes a key factor in eventual food choices.

Breastfed children’s early dietary preferences may translate to greater fruit and vegetable consumption in later childhood. Studies of children from 2 to 13 years old have found that those who were breastfed eat more fruits and vegetables than their formula-fed peers, even after adjusting for key confounders (14–21). Although the strength of causal inference is limited by the observational nature of these studies, this finding has been repeated across diverse cohorts with different food cultures, supporting breastfeeding as a plausible mechanism in flavor learning and increased fruit and vegetable consumption in children.

### Complementary Feeding

Exclusive breastfeeding is generally recommended until 4–6 months of age, at which time infants should begin to consume solid foods in addition to breastmilk (22). This transition, called complementary feeding, ensures that nutritional requirements no longer met by exclusive milk intake are satisfied with food. During this time, acceptance of foods continues to be shaped by repeated exposure to those foods (1, 5). Many experimental studies have demonstrated this to be the case for fruits and vegetables (5, 22). Variety can also promote infants’ acceptance of new foods (11). Infants who were fed various vegetables not including carrots for 9 days ate significantly more carrots and were more accepting of a new food than infants who were only fed potatoes (23). Timing of exposure to new foods also influences infants’ food acceptance. The earlier the introduction to vegetables during complementary feeding, the greater the acceptance of novel vegetables, as assessed by a parent-rated scale of four attributes of the infant’s reaction to the food (12). It is challenging to increase children’s acceptance of fruits and vegetables after toddlerhood (5, 22), making early intervention most promising for improving child diet.

### Parent Feeding Practices

Parents play a crucial role in shaping food preferences, especially in early childhood (24). Their choices of what to serve exert influence on their children’s food preferences (1, 24, 25) because children’s familiarity with a food may be as influential as any particular taste. Given the option of three types of tofu (sweet, salty, or plain), preschoolers preferred the version with which they were familiar (26). How parents’ pair foods can also impact preferences; serving target foods with preferred flavors, such as vegetables with dip, can increase vegetable preference (27).

The social and emotional context of food also influences preferences. Rewarding children for eating enough of a disliked food (28) or forcing them to eat a disliked food (24, 25, 29) decreases their preference for the disliked food, perhaps because this reinforces the idea that the disliked food is unpleasant. However, using non-food rewards, like stickers, to encourage children to try a food has been demonstrated to increase tastings of the target food, which is necessary to increase liking of that food (30–32). This may be an effective strategy for parents to increase their children’s consumption of target foods.

Creating a positive emotional atmosphere around a food increases child preference for it. When children were given a food as a reward or paired with adult attention, their preference for that food increased, even though they initially felt neutral about the food (33). Modeling and flavor conditioning may also contribute to positive food behaviors (7). Children’s intake and preferences can be shaped as they learn from and emulate the eating behaviors of adults and peers (1, 7, 24).

Trying to limit children’s preference for unhealthy foods can be complicated. Because children prefer high levels of sweetness and saltiness (25), they tend to enjoy unhealthy foods. Restricting a given food within a child’s sight increases the child’s preference for it (1, 24, 25), making strict restrictions of unhealthy foods that are regular parts of children’s food environments an unsuccessful strategy in curbing their intake. Controlling strategies for changing children’s eating behavior may be counterproductive; less restrictive approaches such as gardening, cooking programs, and free access to fruits and vegetables may be more effective in encouraging their intake (34).

### Media and Environmental Effects

In a systematic review, the Institute of Medicine found strong evidence that television advertisements influence 2–11 year old children’s food and beverage preferences, requests for purchase, and consumption (35). Children 2–7 years old are exposed to 12 ads/day, and that number increases to 21 for 8–12 year olds (36). Of the 50% of all advertising during children’s shows that is food advertising, 34% is for candy and snacks, 28% for cereal, and 10% for fast food (36). Several observational studies have found that television viewing among young children is associated with poor diet (37), even after adjusting for key sociodemographic factors (38). The effect of advertisements has also been demonstrated experimentally; preschoolers exposed to a food commercial during a cartoon were significantly more likely to choose that food product than unexposed preschoolers (39). This strong short-term impact combined with the constant exposure to advertisements amplifies effects on preferences and food choices.

Food advertising is made even more potent through its long-term branding effects. For example, preschoolers preferred McDonald’s-branded food to the exact same non-branded food items, even when the food was baby carrots, which McDonald’s did not sell at the time of the experiment (40). The branding effect was stronger for children with more television sets at home (40). Another study showed children preferred food branded with popular cartoon characters, particularly for energy-dense, nutrient-poor products (41).

The influence of children’s media environment is compounded by their physical environment. The effects of the physical environment are most pronounced in lower-income neighborhoods, which also tend to have the highest rates of diet-related diseases (42, 43). Corner stores and fast food outlets are especially concentrated in these neighborhoods, while residents have less access to grocery stores and supermarkets (42, 43). Even checkout areas of many non-food retail outlets, such as clothing stores, frequently feature unhealthy foods (44). Children typically start requesting their parents buy certain products at 24 months old; 76% of the time this happens in a supermarket and 77% of first requests are for cereal or sweet snacks (45). Parents honor their children’s food requests about half the time (46). Hence, pervasive exposure to unhealthy foods through media and the food environment heavily influences early childhood preferences for those foods, and makes it challenging for caregivers to provide healthier options.

## Implications and Recommendations for Nutrition Policy and Programs

With food preferences in mind, the following two major areas are paramount for improving diet quality: (1) *Early exposures*: repeated exposure to the flavors of healthy foods is a key, although not the only, mechanism for establishing healthy preferences, especially during the critical period beginning *in utero*, through lactation, and lasting into early childhood; the most learning is required for foods with sour and bitter tastes to which infants are innately averse, such as some fruits and vegetables; and (2) *Environmental exposures*: the social environment around foods, influenced by advertisements, parents, peers, and food access, plays a key role in children’s food preferences; unhealthy foods that cater to children’s innate preferences for sweet and salty tastes are omnipresent and heavily advertised in the modern food environment, while availability of healthy choices is more limited, particularly for low-income populations. Figure 1 summarizes key points of intervention to improve early exposures and environmental exposures. Coordinated efforts across these areas may be required for significant impacts on food preferences, as has been the model followed by successful intervention efforts for childhood obesity-related behaviors (47–49). The following discussion focuses on recommendations for the United States.

### Macro Environment: Policy

Policies such as paid maternity leave can facilitate breastfeeding, a key mechanism for flavor learning. In a study of Danish mothers, who typically take 9–12 months of maternity leave, breastfeeding lasted 41.1 weeks on average (50). Meanwhile, in Greece and the Netherlands, where policy allows 17 and 16 weeks for maternity leave, respectively, breastfeeding lasted an average of 15.2 and 20.7 weeks, respectively (50). Paid maternity leave promotes breastfeeding among mothers in the United States, yet this is the only developed country that does not guarantee paid leave (51). Notably, only 33% of infants in the United States are breastfed for as long as the Danish average (52).

Food prices are an important policy driver of food choices (43). For instance, sugar-sweetened beverage taxes have led to reduced consumption in the United States (53) and Mexico (54, 55). Alongside, policies for incentives and restrictions within food assistance programs may be implemented. Changes to the government-sponsored Supplemental Nutrition Assistance Program (SNAP) for low-income individuals would reach children, as nearly 70% of SNAP participants are families with children (56). A recent study among low-income participants not enrolled in SNAP showed that dietary improvements were greatest for those randomized to receive food assistance with a financial incentive for fruits and vegetables and a restriction on purchasing unhealthy foods such as sugar-sweetened beverages and candy, compared with not having incentives or restrictions (57). A randomized trial of incentives for fruits and vegetables within SNAP also found dietary improvements among those receiving incentives (58). Although the majority of SNAP participants support removing unhealthy foods from SNAP eligibility (59), and similar restrictions are already in place in the Special SNAP for Women, Infants, and Children, proposals to implement restrictions within SNAP have been denied (59). Price-related policies may translate to changes in preference because preferences are shaped by repeated exposure and familiarization (e.g., incentivized healthy foods are more likely to be purchased) and because these policies may reshape children’s food environments (e.g., sugar-sweetened beverage taxes may lower availability in children’s homes). A recent meta-analysis supports the efficacy of subsidies to increase intake of healthful foods and taxation to reduce intake of unhealthful foods (60). Future studies should explore whether price incentives and restrictions change children’s food preferences through these mechanisms.

A final policy approach to positively influence food intake and preferences is limiting food marketing to children. However, developing policies to reduce food advertising to children has been slow (61) and has not led to significant changes in advertising (62). More work in implementing and enforcing policies is required to find effective ways to limit food advertising to children.

### Macro-Environment: Food System

Changes in the food system may limit children’s intake and familiarization with unhealthy foods high in salt and sugar. Processed and restaurant foods are the primary sources of sodium (63, 64) and sugar (65, 66) in the diets of children and adults. Even foods specifically made for infants and toddlers are often high in sugar or sodium (67). Reformulating these foods is feasible; Wal-Mart, for example, has reduced the sodium content of its bread by 16% and its tortillas by 9% (68). Similarly, reducing salt and sugar in restaurant foods, especially those served to children, may allow the next generation of children to become familiar with these foods and develop healthier taste preferences.

### Community

Initiatives in community settings such as hospitals and workplaces can support breastfeeding. The Baby-Friendly Hospital Initiative has been effective for healthcare providers to improve breastfeeding initiation and exclusivity (69). Some workplaces have adopted breastfeeding promotion efforts such as providing a lactation space (70), which is important given the evidence that returning to work impedes breastfeeding (71). These workplace efforts can be effective (70), but they are not prevalent enough; in 2009, 75% of employers in the United States lacked a lactation/mothers’ room (72).

Community initiatives to improve the food environment are also promising, but more research is needed. While there is evidence that farmers market use is positively associated with fruit and vegetable consumption among low-income consumers (73, 74), there is still need for more evaluations across diverse geographies and populations (75, 76). A common challenge is that many farmers markets lack the required equipment to accept SNAP as payment, which has prompted the recommendation to subsidize the equipment for farmers market vendors (76). Others have attempted to work within the existing food infrastructure used by low-income families, such as corner stores, by increasing fruit and vegetable availability or rearranging products within the store to promote healthier purchases (77, 78). These efforts have had mixed success in improving dietary quality (77, 78). Some strategies to change supermarkets, such as promoting healthy foods with signs and sales, have increased purchases of those items, but have been limited in improving diet quality (79).

Daycare (80), preschool (81), and restaurants (82) are other community environments that may influence children’s diets. In restaurants, unhealthy items in children’s meals can be switched for healthier ones, which can lead to healthier ordering without reducing restaurant revenue (82). However, it can be challenging to engage restaurants in creating healthier menu items. For instance, leaders of the Shape Up Somerville program found that fears of profit loss and lack of time impeded menu changes (83).

For childcare and preschools, it is possible to change not only the foods served, but *how* they are served, thus capitalizing on the important social and emotional determinants of food preferences. Compliance with recommended feeding practices, such as eating the same foods as children and encouraging them to eat the foods (84), is variable (85, 86), and providers may not even be aware of them: in Rhode Island almost 70% of providers in family childcare homes had nutrition training three or fewer times in the past 3 years, despite finding it useful and expressing a desire for more training (87). Training has been demonstrated to improve children’s diet quality in childcare centers, making this a promising approach (87). In addition, serving both fruit and vegetables to children in childcare centers—rather than just one of the choices—is associated with higher produce intake (88). Future studies should investigate whether recommendations for increasing provider training and offerings of healthy foods translate to changes in preferences.

### Parents

Parental influence on food preferences begins with decisions, constrained though they are by societal forces, about their own food choices, breastfeeding, and what and how they feed their children (24). Lifestyle counseling can increase vegetable consumption among some subsets of pregnant women (89), but not others (90). For nursing women, diet counseling increased fruit consumption, but not vegetable consumption (91). The evidence of health benefits from healthy diets through the life-course warrants its recommendation during pregnancy, while recognizing the importance of addressing social determinants. Similarly, individual-focused education efforts to promote breastfeeding have small effects (92, 93), highlighting the importance of social determinants of breastfeeding. Systematic reviews have documented that interventions for parent feeding practices (94) and child diet (94, 95) have promise. Suggested parental strategies to increase vegetable intake during early childhood include repeated exposure, modeling, and incentivizing tasting with non-food rewards (96). Not only do early childhood diet interventions match the timing for food preference development, they tend to be more effective than interventions with older children (95).

## Conclusion

The multifactorial origins of food preferences require a similarly multifaceted ecological framework to examine their implications for public health. At all levels, efforts must be taken to promote breastfeeding as well as children’s access to and consumption of healthy foods to create stronger preferences for these foods. Concurrently, it is vital to limit exposures to unhealthy foods with innately preferred sweet and salty tastes that lead to poor overall diet quality. We recommend: (1) implementing policies for paid maternity leave, as recommended by the United Nations’ International Labor Organization since 1952 (97), and establishing breastfeeding-friendly policies in healthcare and workplaces, (2) changing food retail environments to expand geographic and financial access to healthy foods while reducing the ubiquity of unhealthy foods, (3) limiting food marketing to children, (4) reformulating restaurant and processed foods, and (5) training parents, preschool teachers, and childcare providers in appropriate feeding practices that can promote healthy food preferences. In addition to the established benefits of these efforts on improving diet quality and preferences for healthy foods, they have the potential to prevent chronic conditions and improve overall health.

## Author Contributions

JB conceptualized the topic, researched and analyzed the background literature, and wrote the manuscript, including interpretations. QA and EL researched and analyzed the background literature and wrote portions of the manuscript, including interpretations. MT and JM provided substantial scholarly guidance on the conception of the topic, manuscript draft and interpretation and revised the manuscript critically for intellectual content. All the authors approved the final version of the manuscript, ensured the accuracy and integrity of the work, and agreed to be accountable for all aspects of the work.

## Conflict of Interest Statement

The authors declare that the research was conducted in the absence of any commercial or financial relationships that could be construed as a potential conflict of interest.
